# Oxidative Homeostasis in Follicular Fluid and Embryo Quality—A Pilot Study

**DOI:** 10.3390/ijms26010388

**Published:** 2025-01-04

**Authors:** Ana Jeremic, Mladenko Vasiljevic, Zeljko Mikovic, Zoran Bukumiric, Petar Simic, Tamara Stanisavljevic, Tatjana Simic, Tatjana Djukic

**Affiliations:** 1 University Clinic for Gynecology and Obstetrics “Narodni Front”, 11000 Belgrade, Serbia; ana.ivf.ana@gmail.com (A.J.); mladenkovas@icloud.com (M.V.); mikovic.zeljko@gmail.com (Z.M.); simicp93@gmail.com (P.S.); 2 Faculty of Medicine, University of Belgrade, 11000 Belgrade, Serbia; zoran.bukumiric@med.bg.ac.rs (Z.B.); tamara.stanisavljevic@med.bg.ac.rs (T.S.); 3 Institute of Medical Statistics and Informatics, 11000 Belgrade, Serbia; 4 Institute of Medical and Clinical Biochemistry, 11000 Belgrade, Serbia; 5 Center of Excellence for Redox Medicine, 11000 Belgrade, Serbia; 6 Serbian Academy of Sciences and Arts, 11000 Belgrade, Serbia

**Keywords:** infertility, follicular fluid, embryo quality, oxidative stress, malondialdehyde, 8-hydroxy-2′-deoxyguanosine, thiol groups, superoxide dismutase, glutathione peroxidase, glutathione transferase

## Abstract

The objective of this study was to measure the different redox biomarker levels within the follicular fluid (FF) and evaluate correlations with embryo quality using the one follicle–one oocyte/embryo approach. The prospective study included 54 women (average age 34.6 ± 3.0 years). Out of the 235 mature metaphase II cells that underwent intracytoplasmic sperm injection, fertilization was achieved in 177 cells, producing 92 Grade I embryos, 26 Grade II embryos, 39 Grade III embryos, and 20 Grade IV embryos. The activities of antioxidant enzymes, superoxide dismutase, glutathione peroxidase, and glutathione transferase were significantly higher in the group consisting of lower-quality (Grades II–IV) embryos in comparison with top-quality (Grade I) embryos (*p* = 0.011; *p* = 0.021; *p* = 0.008, respectively). The concentration of oxidative stress markers, malondialdehyde, 8-hydroxy-2′-deoxyguanosine, and thiol groups was significantly increased in the group with lower-quality embryos (Grades II–IV) compared to top-quality embryos (0.027; 0.018; 0.021, respectively). Furthermore, a significant positive correlation between each oxidative marker and the activities of antioxidant enzymes was observed (*p* < 0.001). According to our findings, the best embryos and, consequently, better in vitro fertilization outcomes are linked to low levels of oxidative stress and low antioxidant enzyme activity.

## 1. Introduction

Millions of couples worldwide are affected by infertility, i.e., the inability to conceive after 12 months of regular and unprotected intercourse [1]. Oxidative stress (OS) has been identified as a major factor in the pathophysiology of both male and female infertility [2,3,4]. It is defined as an “imbalance between oxidants and antioxidants in favor of the oxidants, leading to a disruption of redox signaling and control and/or molecular damage” [5]. The excess of pro-oxidants, such as reactive oxygen species (ROS), reactive nitrogen species (RNS), etc., is surmounted by prompt antioxidant defense systems [6]. The vast majority of studies imply that ROS significantly accelerates the progressive qualitative and quantitative worsening of the ovarian oocyte reserve, leading to decreased oocyte competence and reduced fertility [7].

The follicular fluid provides a microenvironment for oocyte development and plays a fundamental role in oocyte performance. Under physiological conditions, antioxidant defense systems scavenge free radicals and prevent the further propagation of their production. Therefore, any imbalance between the antioxidant systems and ROS production in the follicular fluid (FF) may induce oxidative stress, resulting in the abnormal development of the oocyte, which causes damage to the cytoskeleton, DNA, and cell membrane, thus affecting the pregnancy outcome [8,9,10]. A growing body of evidence suggests that a fine balance between ROS and antioxidants is crucial for successful reproductive outcomes both in vivo and in vitro [11,12]. On the other hand, a certain level of reactive oxygen species (ROS) is required to facilitate normal cellular functioning [13]. Thus, physiological ROS levels in the FF of women undergoing in vitro fertilization (IVF) are particularly important due to the additional risk of oxidative stress as a result of external factors. They are needed for the healthy development of oocytes and ovulation during folliculogenesis, luteal regression, successful fertilization, and embryo formation, as well as sperm functions such as oocyte fusion, capacitation, and acrosome reaction [13,14].

Regarding oxidative stress and IVF, there is an agreement between different study groups: oxidative stress increases with the age of a woman, which is related to poor reproductive outcomes [15,16]. Pathological conditions, such as endometriosis and polycystic ovarian syndrome (PCOS), can induce oxidative stress within the follicular environment, which, in turn, increases the level of ROS [17,18,19]. According to Pérez-Ruiz et al., the level of oxidative stress is increased in the controlled ovarian hyperstimulation (COH) cycle compared to the natural ovulation cycle (NC) [20].

Still, the role of free radicals and the potential of oxidative stress byproducts as biomarkers of IVF success are controversial. Therefore, the objective of our study was to investigate the level of oxidative stress biomarkers (malondialdehyde (MDA), 8-hydroxy-2′-deoxyguanosine (8-OHdG) and thiol groups) and antioxidant enzyme activities (superoxide dismutase (SOD), glutathione peroxidase (GPx) and glutathione S-transferase (GST)) in the FF of women undergoing IVF and to assess the association between redox homeostasis in the FF and embryo quality using the one follicle–one oocyte/embryo approach. Furthermore, the correlation between oxidative stress biomarkers and antioxidant enzyme activities was determined.

## 2. Results

**Figure 1:**
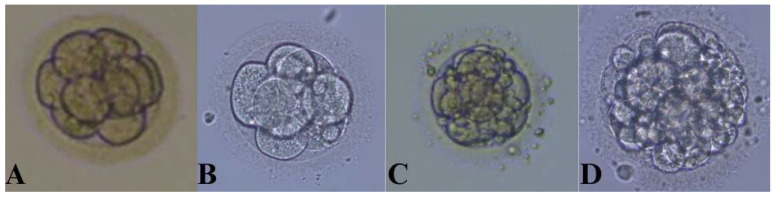
Evaluation of embryo quality on day 3. (**A**) Grade I, (**B**) Grade II, (**C**) Grade III, and (**D**) Grade IV embryo.

This study assessed 235 follicles from 54 women who underwent IVF. Out of the 235 MII cells that underwent ICSI, fertilization was achieved in 177 cells. All mature (MII) oocytes to which the ICSI method was applied and whose development was further followed up to day 3 (D3) were divided into four groups: Grade I embryos (92 embryos)—embryos with ≤10% fragmentation which consisted of 3–4 blastomeres on day 2 and 6–8 blastomeres on day 3 (top-quality embryos); Grade II embryos (26 embryos)—embryos with between 10% and 20% fragmentation which consisted of 3–4 blastomeres on day 2 and 6–8 blastomeres on day 3 (good-quality embryos); Grade III embryos (39 embryos)—embryos with 2–8 blastomeres on day 3 and between 20% and 50% fragmentation (fair-quality embryos); and Grade IV (20 embryos)—embryos with >50% fragmentation (poor-quality embryos) (Figure 1).

The women enrolled in the study had an average age of 34.6 ± 3.0 and an average body mass index (BMI) of 23.0 ± 2.9 kg/m^2^. The mean estradiol level on the day of hCG injection was 7104.7 ± 2772.5 pmol/L. Selected clinical characteristics are presented in Table 1.

To examine whether the disturbance of redox homeostasis is associated with a lower oocyte fertilization rate, markers of the oxidative stress and the antioxidant enzyme activities in follicular fluid were measured in the group where fertilization occurred as well as in the group where fertilization did not occur, as shown in Table 2. No statistically significant differences were found in the concentration of thiol groups (*p* = 0.307), MDA (*p* = 0.935), or 8-OHdG (*p* = 0.504) in those two groups. Comparing the groups where fertilization happened and did not occur, there were also no statistically significant differences in SOD activity (*p* = 0.342), GPx (*p* = 0.194), or GST activity (*p* = 0.256) (Table 2).

**Figure 2:**
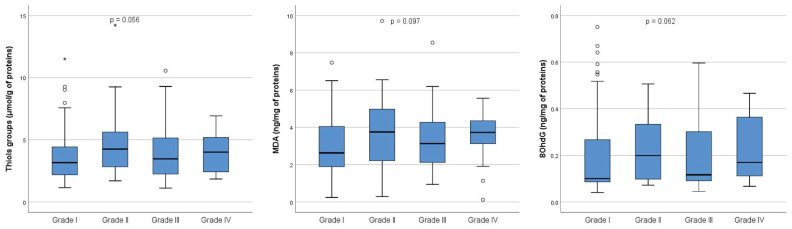
Oxidative stress markers in follicular fluid on the day of oocyte retrieval in 54 patients who underwent controlled ovarian hyperstimulation. The graph displays the distribution of values across each grade. The central horizontal line within each box represents the median (50th percentile). Each box encompasses the interquartile range (25th to 75th percentile), with whiskers extending to the minimum and maximum values within 1.5 times the interquartile range. Outliers are represented by circles, while extreme values are indicated by asterisks. Thiol groups: Grade I (n = 92), median 3.16 (range, 1.15–20.73); Grade II (n = 24), median 4.26 (range, 1.70–58.82); Grade III (n = 39), median 3.47 (range, 1.11–71.99); Grade IV (n = 20), median 4.01 (range, 1.85–6.92). MDA: Grade I (n = 91), median 2.63 (range, 0.24–21.63). Grade II (n = 24), median 3.75 (range, 0.29–11.34); Grade III (n = 38), median 3.13 (range, 0.94–8.55); Grade IV (n = 19), median 3.73 (range, 0.12–5.56). 80hdG: Grade I (n = 91), median 0.10 (range, 0.04–0.75); Grade II (n = 24), median 0.20 (range, 0.07–1.33); Grade III (n = 39), median 0.12 (range, 0.05–7.92); Grade IV (n = 20), median 0.17 (range, 0.07–0.47). The *p*-value refers to overall differences between Grades I, II, III, and IV. Out of the 235 MII cells that underwent ICSI, fertilization was achieved in 177 cells, producing 92 Grade I embryos, 26 Grade II embryos, 39 Grade III embryos, and 20 Grade IV embryos. MDA = malonaldehyde; 8OHdG = 8-hydroxy-2′-deoxyguanosine; n = number of samples (embryos) analyzed for each grade.

Furthermore, a comparative analysis of markers of redox homeostasis in follicular fluid was performed between the four grades of embryos. As shown in Figure 2, there were no overall significant differences in the concentration of thiol groups (*p* = 0.056) between four grades of embryos, nor the concentration of MDA (*p* = 0.097) and 8-OHdG (*p* = 0.062).

**Figure 3:**
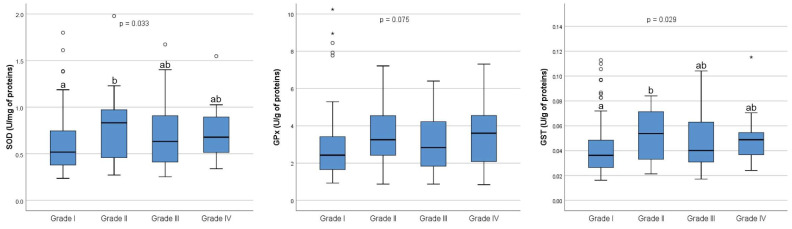
Antioxidant enzyme activities in follicular fluid on the day of oocyte retrieval in 54 patients who underwent controlled ovarian hyperstimulation. The graph displays the distribution of values across each grade. The central horizontal line within each box represents the median (50th percentile). Each box encompasses the interquartile range (25th to 75th percentile), with whiskers extending to the minimum and maximum values within 1.5 times the interquartile range. Outliers are represented by circles, while extreme values are indicated by asterisks. SOD: Grade I (n = 92), median 0.52 (range, 0.24–4.61); Grade II (n = 24), median 0.83 (range, 0.27–10.56); Grade III (n = 38), median 0.63 (range, 0.25–8.46); Grade IV (n = 20), median 0.68 (range, 0.34–1.55). GPx: Grade I (n = 92), median 2.42 (range, 0.93–14.11); Grade II (n = 24), median 3.25 (range, 0.87–20.90); Grade III (n = 39), median 2.82 (range, 0.87–41.22); Grade IV (n = 20), median 3.60 (range, 0.84–7.31). GST: Grade I (n = 92), median 0.036 (range, 0.016–0.302); Grade II (n = 24), median 0.054 (range, 0.021–0.733); Grade III (n = 38), median 0.040 (range, 0.017–0.708); Grade IV (n = 20), median 0.049 (range, 0.024–0.115). The *p*-value refers to overall differences between Grades I, II, III, and IV. Values with different letters (a, b) are significant at *p* < 0.05. Out of the 235 MII cells that underwent ICSI, fertilization was achieved in 177 cells, producing 92 Grade I embryos, 26 Grade II embryos, 39 Grade III embryos, and 20 Grade IV embryos. SOD = superoxide dismutase; GPx = glutathione peroxidase; GST = glutathione-S transferase; n = number of samples (embryos) analyzed for each grade.

As for the SOD activity, an overall statistically significant difference was found among the four grades of embryos (*p* = 0.033). In the post hoc analysis, a statistically significant difference was found only between Grade I and Grade II embryos (*p* = 0.045) (Figure 3). Similarly, an overall statistically significant difference was observed in GST activity among the four grades of embryos (*p* = 0.029). Post hoc analysis revealed a statistically significant difference in GST activity only between Grade I and Grade II embryos (*p* = 0.048). However, no statistically significant differences in GPx activity were found among the four grades of embryos (*p* = 0.075) (Figure 3).

However, because we did not find any significant differences in the activities of antioxidant enzymes or in the concentration of oxidative markers between four grades of embryos, we performed a comparative analysis between the best quality embryos (Grade I) and grouped embryos of Grades II–IV (lower-quality embryos). The embryos were assessed on the 3rd day.

**Figure 4:**
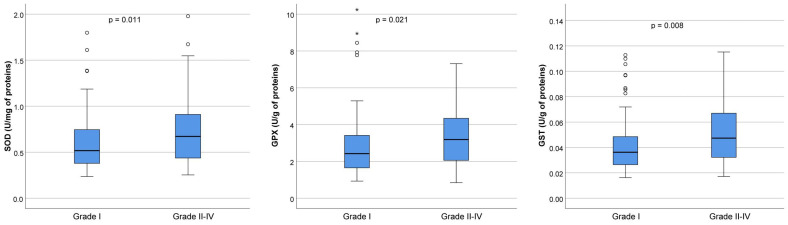
Antioxidant enzyme activities in follicular fluid on the day of oocyte retrieval in 54 patients who underwent controlled ovarian hyperstimulation. The graph displays the distribution of values across each grade. The central horizontal line within each box represents the median (50th percentile). Each box encompasses the interquartile range (25th to 75th percentile), with whiskers extending to the minimum and maximum values within 1.5 times the interquartile range. Outliers are represented by circles, while extreme values are indicated by asterisks. SOD: Grade I (n = 92), median 0.52 (range, 0.24–4.61); Grade II–IV (n = 82), median 0.67 (range, 0.25–10.56). GPx: Grade I (n = 92), median 2.42 (range, 0.93–14.11); Grade II–IV (n = 83), median 3.19 (range, 0.84–41.22). GST: Grade I (n = 92), median 0.036 (range, 0.016–0.302); Grade II–IV (n = 82), median 0.047 (range, 0.017–0.733). The *p*-value refers to overall differences between Grades I, II, III, and IV. Out of the 235 MII cells that underwent ICSI, fertilization was achieved in 177 cells, producing 92 Grade I embryos, 26 Grade II embryos, 39 Grade III embryos, and 20 Grade IV embryos. SOD = superoxide dismutase; GPx = glutathione peroxidase; GST = glutathione-S transferase; n = number of samples (embryos) analyzed for each grade.

The SOD activity was significantly higher in the group containing lower-quality embryos (0.52 U/mg of proteins in Grade I versus 0.67 U/mg of proteins in Grades II–IV, *p* = 0.011). Similarly, the GPx activity was significantly higher in the group with lower-quality embryos (2.42 U/g of proteins in Grade I versus 3.19 U/g of proteins in Grades II–IV, *p* = 0.021). Additionally, the group of embryos with lower quality had considerably higher GST activity (0.04 U/g of proteins in Grade I versus 0.05 U/g of proteins in Grades II–IV, *p* = 0.008) (Figure 4).

**Figure 5:**
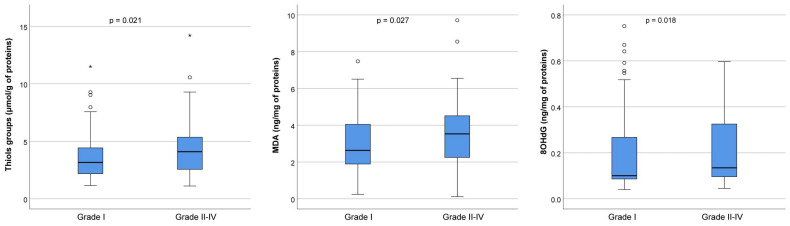
Oxidative stress markers in follicular fluid on the day of oocyte retrieval in 54 patients who underwent controlled ovarian hyperstimulation. The graph displays the distribution of values across each grade. The central horizontal line within each box represents the median (50th percentile). Each box encompasses the interquartile range (25th to 75th percentile), with whiskers extending to the minimum and maximum values within 1.5 times the interquartile range. Outliers are represented by circles, while extreme values are indicated by asterisks. Thiol groups: Grade I (n = 92), median 3.16 (range, 1.15–20.73); Grade II–IV (n = 83), median 4.10 (range, 1.11–71.99). MDA: Grade I (n = 91), median 2.63 (range, 0.24–21.63); Grade II–IV (n = 81), median 3.53 (range, 0.12–11.34). 8OHdG: Grade I (n = 91), median 0.10 (range, 0.04–0.75); Grade II–IV (n = 83), median 0.13 (range, 0.05–7.92). The *p*-value refers to overall differences between Grades I, II, III, and IV. Out of the 235 MII cells that underwent ICSI, fertilization was achieved in 177 cells, producing 92 Grade I embryos, 26 Grade II embryos, 39 Grade III embryos, and 20 Grade IV embryos. MDA = malonaldehyde; 8OHdG = 8-hydroxy-2′-deoxyguanosine; n = number of samples (embryos) analyzed for each group.

To detect oxidative stress levels, we measured the concentration of thiol groups, MDA, and 8-OHdG in FF in the best-quality embryos (Grade I) and lower-quality embryos of Grades II–IV, as shown in Figure 5. In particular, the concentration of thiol groups was significantly increased in the group with lower-quality embryos (3.2 μmol/g of proteins in Grade I versus 4.1 μmol/g of proteins in Grades II–IV, *p* = 0.021), as well as MDA (2.6 ng/mg of proteins in Grade I versus 3.6 ng/mg of proteins in Grades II–IV, *p* = 0.027) and 8-OHdG (0.10 ng/mL of proteins in Grade I versus 0.13 ng/mL of proteins in Grades II–IV, *p* = 0.018).

**Figure 6:**
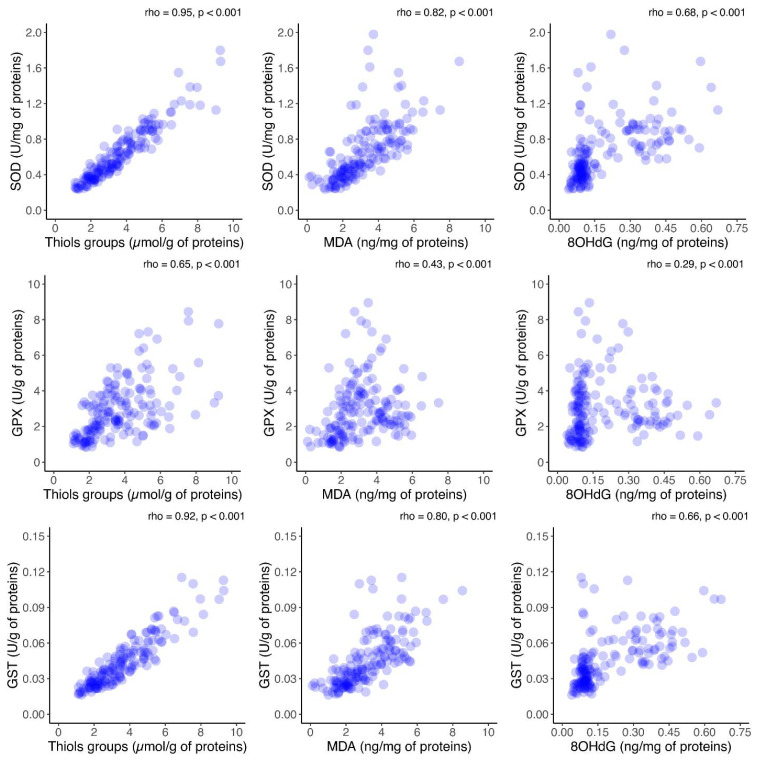
Scatter plot analysis of the correlation between oxidative stress markers and antioxidant enzyme activities in follicular fluid on the day of oocyte retrieval in 54 patients who underwent controlled ovarian hyperstimulation. Outliers are not shown in the graph for a better visualization of the correlation. SOD = superoxide dismutase; GPx = glutathione peroxidase; GST = glutathione-S transferase; MDA = malonaldehyde; 8OHdG = 8-hydroxy-2′-deoxyguanosine.

Furthermore, to assess the relationship between oxidative stress and antioxidant activity, the correlations between antioxidant enzyme activity and oxidative stress markers in FF was investigated. A significant positive correlation between each oxidative marker and the activities of antioxidant enzymes was observed (*p* < 0.001), as shown in Figure 6. Additionally, the correlation between antioxidant enzymes and oxidative stress biomarkers in the FF was investigated in the group consisting of Grade I embryos (Figure S1, Supplementary files). The same analysis was performed in the group consisting of Grade II–IV embryos (Figure S2, Supplementary files). As for the results of the analysis presented in those two figures, they are similar with the ones obtained and presented in Figure 6.

## 3. Discussion

During in vitro fertilization (IVF), the data gathered from the examination of the follicular fluid (FF) helped to determine the oocyte competence for a high-quality embryo. Therefore, the assessment of the biochemical composition of the FF has a significant role as a non-invasive predictive method in IVF processes [21].

The activity of the antioxidant enzymes SOD, GST, and GPx and the indicators of proteins, lipids, and DNA oxidative modifications in the FF of patients undergoing IVF and their correlation with embryo quality were analyzed in this study in an attempt to shed light on the mechanisms of oxidative stress during the assisted reproductive technology (ART) process and the impact of disturbed redox homeostasis on the outcome of ART.

To our knowledge, this is the first prospective study to examine these six biochemical parameters in FF by using the one follicle–one oocyte/embryo approach in a very homogeneous group of normoresponders. In our study, the activity of antioxidant enzymes, SOD, GPx, and GST was significantly higher in the group consisting of Grade II–IV embryos in comparison with top-quality embryos. The concentration of oxidative stress markers, MDA, 8-OHdG, and thiol groups, was significantly increased in the group with lower-quality embryos (Grades II–IV) compared to top-quality embryos. Furthermore, a significant positive correlation between each oxidative marker and the activities of antioxidant enzymes was observed.

Previous studies have shown that poor-quality oocytes are characterized by high mitochondrial DNA damage and aneuploidy [13]. 8-hydroxy-2′-deoxyguanosine (8-OHdG) has been widely used as a biomarker for oxidative stress and is one of the most important markers of DNA damage [22]. Indeed, in our study, a significantly increased concentration of 8-OHdG was observed in the group with lower-quality embryos, which is in concordance with previously published data. Varnagy et al. showed that 8-OHdG has a negative effect on the number of good-quality embryos [23]. Nishihara et al. investigated the correlation between FF oxidative stress markers (8-OHdG) and antioxidant status (total glutathione) in relation to intracytoplasmic sperm injection (ICSI) outcomes and embryo transfer rates. What they found is that patients with endometriosis had the highest level of 8-OHdG in their FF [24]. Additionally, Mukheef et al. investigated the influence of this oxidative marker on the intracytoplasmic sperm injection (ICSI) outcome and observed a significant negative correlation of 8-OHdG with the number of obtained oocytes, metaphase II oocytes, fertilization, embryos, and embryos of good quality. They concluded that 8-OHdG has a negative impact on the outcome of ICSI and is higher in women who have not become pregnant [8]. The analysis of Nori and Helmi emphasized the significant differences in the numbers of retrieved oocytes and 8-OHdG levels in the follicular fluid based on the cause of infertility. The highest concentration of FF 8-OHdG was observed in the endometriosis groups, followed by the polycystic ovary syndrome group. Furthermore, 8-OHdG levels in the follicular fluid were significantly increased in non-pregnant women compared to pregnant women [25]. Da Broi et al. proposed follicular 8-OHdG as a predictive marker for clinical pregnancy in patients with endometriosis [17]. Interpreting these results is made more difficult by the absence of specific pro-oxidant and antioxidant biomarker concentrations in physiological settings [26].

MDA is the lipid peroxidation decomposition product generated by free radical damage to unsaturated fatty acids. MDA levels in the FF are considered a prognostic factor in IVF processes [27,28]. What was found in our study was a significantly increased MDA concentration in the group with lower-quality embryos in comparison with Grade I embryos. In line with our findings, a negative correlation was established between the follicular MDA level and the number of top-quality embryos in the study of Uppangala et al. [9]. This was not the case in other randomized studies. Pasqualotto et al. and Fujimoto et al. found no correlation between lipid peroxidation product levels in follicular fluid and embryo quality in their studies [29,30]. Nuñez-Calonge et al. found that young women with low ovarian response had a decreased activity of GPx, GR, and GST in comparison with oocyte donors and high responders. This decreased antioxidant enzyme activity was followed by increased concentrations of lipid peroxides, MDA, and 4-hydroxyalkenals [31].

The main sites of action of ROS on proteins are free thiol groups that do not participate in the formation of disulfide bonds, and they are most often used as markers of oxidative damage to proteins [14]. To detect the oxidative modifications of proteins, the concentration of the thiol groups was measured in our study. A significantly lower concentration was found in the group with excellent embryos in comparison with Grade II–IV embryos.

Antioxidant enzymes responsible for scavenging ROS and preventing the occurrence of oxidative stress in cells are also found in the FF. The exact mechanisms of their protection of oocytes from oxidative stress in vitro conditions have not yet been fully elucidated [2].

Superoxide dismutase is an antioxidant enzyme considered the first line of defense against free radicals [32]. Previously published data have shown that SOD activity decreases with age and that low SOD activity negatively affects the outcome of the IVF process [18]. In our study, the SOD activity was found to be significantly higher in the group of low-quality embryos compared to Grade I embryos. These results are different from those observed by Seleem et al., who found that SOD activity in the FF does not affect the fertilization rate and embryo quality [33]. In a manner similar to oxidative stress biomarkers, an adaptive response to SOD activity was found in our study. Indeed, SOD activity was statistically significantly positively correlated with each oxidative marker. The higher activity of an antioxidant enzyme may be a compensatory regulation in response to increased oxidative stress.

GPx and GST are the other two enzymes that belong to the antioxidant defense system [34]. Our results have shown that, similarly to SOD, the activities of these enzymes were significantly increased in the group with Grade II–IV embryos compared to top-quality embryos. Our results are in concordance with those of Meijide et al., who found significantly lower GST activity in the mature oocytes compared to the immature ones [35]. Further, those women who achieved pregnancy had increased levels of total antioxidant capacity and GpX and decreased levels of ROS, MDA, glutathione reductase, SOD, and GST [28,36,37]. In our study, a significant positive correlation between each oxidative marker and both GPx and GST activities was observed. However, of all the antioxidant enzymes that have been related to reproduction in vivo and in vitro, GST has been the least studied.

Based on the correlation between markers of oxidative stress and antioxidant enzyme activities found in this study, it seems reasonable to assume that there is a fine-tuning of enzyme activity dependent on the level of free radical production, possibly by the Nrf2-mediated oxidative-stress-response pathway. MDA, 8-OHdG, and thiol group levels were positively correlated with the activities of antioxidant enzymes and exhibited higher concentrations in the group of lower-quality embryos (Grade II–IV). It seems plausible to conclude that low levels of thiol groups in the FF are associated with the development of high-quality embryos. It appears that the level of thiol groups in the FF, as a factor in oocyte maturation and fertilization, might be interpreted as a biomarker of so-called oxidative eustress, which corresponds to the physiological changes in thiol group oxidation, most frequently induced by physiological variations in H_2_O_2_ levels [38]. The most common target of reactive oxygen species-mediated signaling is the reversible thiol oxidation of cysteines in specific redox-sensitive proteins, resulting in protein disulfide bond formation [39]. In the early stages of oocyte development, when variations in O_2_ tension and spikes in ROS production are common, several of these signaling pathways and targets have homeostatic functions [40]. Thus, our findings that lower thiol group levels are found in Grade I embryos support the idea that some level of redox signaling occurs during fertilization. This highlights the need for caution regarding supplementation with different antioxidants, as they may disrupt redox signaling.

According to our findings, the best embryos and, consequently, better IVF outcomes are linked to low levels of oxidative stress and low antioxidant enzyme activity. These findings may help IVF practitioners to create more successful methods for choosing oocytes and embryos with higher chances of success and pave the way for the implementation of the non-invasive biochemical analysis of follicular fluid. In addition, it emphasizes the importance of the one follicle–one oocyte/embryo approach. It is plausible to assume that the individual estimation of oxidative stress markers and antioxidant enzymes simultaneously in the follicular fluid is needed because no single oxidative stress biomarker has been suggested, and the mechanism by which antioxidants contribute to successful ICSI outcomes has not been clarified.

The major limitation of our research is the small sample size used due to the rigorous inclusion/exclusion criteria. Also, this study was designed according to the one follicle–one oocyte/embryo approach. Furthermore, according to the literature, embryo transfers on day 5 (blastocysts stage) have a higher pregnancy rate than embryos transferred earlier (day 2 or 3). The development of embryos is a genetically dynamic process, and the zygote genome activation begins approximately at the four- to eight-cell stage, which indicates that the good morphological quality of embryos on day 3 is not a guarantee that the said embryo will reach the blastocyst stage. Keeping that in mind, we recommend expanding the research on prolonged embryo cultivation to the blastocyst stage (day 5/6), as this would be more reliable.

## 4. Materials and Methods

This prospective study included 54 women (average age 34.6 ± 3.0 years) treated in the Gynecology and Obstetrics Clinic “Narodni Front” between March and June 2023. The enrolment inclusion criteria were as follows: infertile women between the ages of 30 and 38 with a preserved ovarian reserve and normal response to gonadotropin stimulation, an anti-Müllerian hormone (AMH) level greater than 1, a number of antral follicles between 5 and 20, a body mass index (BMI) < 25, having undergone three intrauterine insemination procedures in conjunction with ovulation induction to achieve pregnancy and whose male partner had a normal spermogram according to the criteria of the World Health Organization, as well as follicular fluids from which mature-metaphase II (MII) oocytes could be obtained, which were fertilized by means of the intracytoplasmic sperm injection (ICSI) method [41,42]. The exclusion criteria were as follows: patients with endometriosis or polycystic ovary syndrome (PCOS); patient oocytes obtained from hemorrhagic follicular fluids and follicular fluids containing a flushing medium, as well as follicular fluids from which oocytes were degenerate, immature germinal vesicles (GV) or immature metaphase I (MI) cells; and oocytes destined for the method of conventional in vitro fertilization. A total number of 65 patients were recruited, and informed consent was obtained to participate in the study. Of these 65 women, 54 were enrolled in this study because 11 of them did not meet the criteria due to hemorrhagic follicles, a flushing medium content, or no MII cells destined for ICSI being obtained from follicular fluids. From the 54 patients who entered the study (excluding the cells from hemorrhagic follicles or follicular fluids that contained the flushing medium, as well as those that were selected for the conventional IVF method), 300 cells were obtained. After oocyte denudation from the surrounding cumulus cells, 235 mature MII cells were ultimately isolated and underwent the ICSI method. The remaining 65 cells, since they were not MII, were excluded from this study. This study was designed to assess the association between redox homeostasis in follicular fluid and embryo quality using the 1 follicle–1 oocyte/embryo approach. The activities of the antioxidant enzymes SOD, GPx, and GST, as well as the level of lipid peroxidation (MDA), DNA damage (8-OHdG), and the concentration of thiol groups, were measured in the follicular fluid of each oocyte that was subjected to the intracytoplasmic sperm injection (ICSI) method, and embryo quality on the third day of cultivation were individually monitored.

### 4.1. Ovarian Stimulation Protocol

A short gonadotropin-releasing hormone (GnRH) antagonist protocol was used to stimulate ovulation in patients [43,44]. During ovulation stimulation, hormone levels were monitored, including estradiol (E2), the luteinizing hormone (LH), and progesterone (P), while ultrasound folliculometry was also performed (follicle size and endometrial thickness). To stimulate ovulation, the patients received between 75 and 225 IU of highly purified urinary gonadotropin in combination with 75 IU of the recombinant follicle-stimulating hormone (FSH) daily. Cetrorelix or ganirelix at a concentration of 0.25 mg/mL was used when the leading follicle was ≥ 14 mm and until the end of the stimulation. When 3 follicles ≥ 17 mm were verified by ultrasound, choriogonadotropin alpha (Ovitrelle, Merck Serono, Darmstadt, Germany) was used to trigger final oocyte maturation in all patients. The transvaginal aspiration of follicles was performed under ultrasound control 35 h after the administration of choriogonadotropin alpha.

### 4.2. Collection and Preparation of Follicular Fluid

Individual follicular fluids during oocyte retrieval were aspirated and collected into separate tubes. Each oocyte was assigned a unique identification number, which was identical to that of the FF from which it was obtained. FF was not collected from follicles containing blood and flushing media or from follicles with no oocytes or containing MI, GV, or degenerated oocytes. FF aspirated from follicles during oocyte retrieval was centrifugated at 3000× *g* for 10 min, and clarified FF without various cellular components was stored at −80 °C.

### 4.3. Evaluation of Embryo Quality

Out of the 235 MII cells that underwent ICSI, fertilization was achieved in 177 cells. The remaining 58 cells did not achieve fertilization. The development of all mature (MII) oocytes to which the ICSI method was applied was followed up to day 3 (D3) when the embryo transfer was performed. Since the embryo transfer occurred on the 3rd day, we divided the embryos into four groups based on their morphology on day 3 of cultivation: Grade I embryos (92 embryos)—embryos with ≤10% fragmentation which consisted of 3–4 blastomeres on day 2 and 6–8 blastomeres on day 3 (top-quality embryos); Grade II embryos (26 embryos)—embryos with between 10% and 20% fragmentation which consisted of 3–4 blastomeres on day 2 and 6–8 blastomeres on day 3 (good-quality embryos); Grade III embryos (39 embryos)—embryos with 2–8 blastomeres on day 3 and between 20% and 50% fragmentation (fair-quality embryos); and Grade IV (20 embryos)—embryos with >50% fragmentation (poor-quality embryos). A representative example of Grade I–IV embryos is presented in Figure 1. For comparison purposes, the retrieved oocytes were divided into two groups: Group I consisted of Grade I embryos (top-quality embryos), and Group II consisted of embryos belonging to Grades II, III, and IV (lower-quality embryos).

### 4.4. Biochemical Analyses

Measurements of the lipid peroxidation marker (MDA) and the DNA damage marker (8-OHdG) in FF were performed using commercially available assays (ELISA Kit, Elabscience, Houston, TX, USA, catalog numbers E-EL-0060 and E-EL-0028, respectively) and were expressed as ng/mg protein. The concentration of thiol groups in FF was determined spectrophotometrically according to the method of Jocelyn et al. and was expressed as μmol/g of protein [45]. The method used by Jocelyn et al. is based on reactions of thiols with Ellman’s reagent (5,5′-dithiobis-2-nitrobenzoic acid, DTNB). Protein thiol groups react with DTNB, cleaving the disulfide bond to give 2-nitro-5-thiobenzoate (TNB), and yield a yellow color. Since sunlight can reduce DTNB, all reactions were performed in a dark place or were protected from sunlight. DTNB (4 mg/mL) was dissolved in a 0.1 M sodium phosphate-buffer solution at pH 8.0. A set of bovine serum albumin standards was prepared. The test tubes contained 150 µL of DTNB, 450 µL of sodium phosphate buffer, and 300 µL of the standard or sample. The test tubes were incubated at room temperature for 15 min, and the optical absorbance was measured at 412 nm. The absorbance values obtained for each standard were plotted versus bovine serum albumin concentrations to obtain the calibration curve. The concentration of thiol groups in the sample was obtained using the calibration curve. The activity of SOD in the FF was determined spectrophotometrically according to the Misra–Fridovich method expressed as U/mg protein, while the activity of GPx in the FF was determined according to the method used by Gunzler et al. and expressed as U/g protein [46,47]. The method for determining the SOD activity uses the inhibition of the auto-oxidation of epinephrine to adenochrome by SOD in an alkaline environment. Briefly, 100 μL of FF and 1380 μL of the buffer (50 mM sodium bicarbonate with 1 mM EDTA, pH 10.2) were mixed, and then 100 μL of epinephrine was added. The reaction was monitored kinetically and was started with the addition of epinephrine, and the change in absorbance was monitored at 480 nm. The activity unit of SOD is the amount of the enzyme needed to cause a 50% inhibition of the reaction at a maximum increment of absorbency of 0.025 u/min. As for Gpx activity, this method is based on the reduction in t-butyl hydroperoxide (t-BOOH) by GPx. Oxidized glutathione (GSSG), produced upon the reduction in t-BOOH, is recycled to its reduced state (GSH) by the enzyme glutathione reductase (GR) in the presence of NADPH. The oxidation of NADPH to NADP+ is accompanied by a decrease in absorbance at 340 nm, providing a spectrophotometric means for monitoring GPx enzyme activity. The reaction mixture contained 870 µL of 50 mM Tris Buffer, pH 7.6, 20 µL of GR (50 U/mL), 20 µL of GSH (0.15 M), 20 µL of NADPH (8.4 mM), and 50 µL of FF. The reaction began after adding 20 µL of t-BOOH (30 mM). One unit of GPx activity was reported as mmol NADPH oxidized/min, assuming 6.22 × 103/L/mol/cm to be the molar absorbency of NADPH at 340 nm. GST activity in FF was measured spectrophotometrically using the methods of Habig et al. and was expressed as U/g protein [48]. GST activity was measured spectrophotometrically at 340 nm by monitoring the rate of glutathione and 1-chloro-2,4-nitrobenzene (CDNB) conjugate formation. The reaction mixture contained 1750 µL of a 100 mM phosphate buffer, pH 6.5, with 1 mM EDTA, 100 µL of 20 mM glutathione, and 100 µL of 20 mM CDNB. The reaction began by adding 50 µL of FF.

### 4.5. Statistical Analysis

Depending on the type of variables and the normality of the distribution, results are presented as the frequency (percent), median (range), or mean ± standard deviation. For data with a non-normal distribution, the Mann–Whitney U test was used to test differences between groups, while the Kruskal–Wallis test with Bonferroni correction for multiple comparisons was used to compare more than two groups. The correlation between oxidative stress markers and antioxidant enzyme activity in the FF was estimated using Spearman’s correlation coefficient. All *p*-values less than 0.05 were considered significant. Statistical data analysis was performed using IBM SPSS Statistics 22 (IBM Corporation, Armonk, NY, USA) and R-4.0.0 software (The R Foundation for Statistical Computing, Vienna, Austria).

## Figures and Tables

**Table 1:** Clinical characteristics of the women who underwent controlled ovarian hyperstimulation.

Variable	Patients (n = 54)
Age, mean ± SD ^1^	34.6 ± 3.0
Body mass index (kg/m^2^), mean ± SD	23.0 ± 2.9
Smoking, n (%)	
No	35 (64.8)
Yes	19 (35.2%)
Number of stimulation days, median (range)	10.0 (8.0–14.0)
Number of MII oocytes, median (range)	4.0 (1.0–10.0)
AMH (ng/mL), median (range)	3.1 (1.0–9.0)
E2 peak level (pmol/L), mean ± SD	7104.7 ± 2772.5

^1^ SD = standard deviation; n = number of participants; AMH = anti-Mullerian hormone; E2 = estradiol.

**Table 2:** Antioxidant enzyme activities and oxidative stress markers in follicular fluid on the day of oocyte retrieval in patients who underwent controlled ovarian hyperstimulation.

Variables	Fertilized n = 177	Unfertilized n = 58	*p*-Value
SOD, U/mg of proteins	0.58 (0.24–10.46)	0.63 (0.29–4.28)	0.342
GPX, U/g of proteins	2.76 (0.84–41.22)	3.10 (0.98–20.69)	0.194
GST, U/g of proteins	0.40 (0.02–0.73)	0.41 (0.02–0.23)	0.256
Thiol groups, μmol/g of proteins	3.47 (1.11–71.99)	4.1 (1.1–72.0)	0.307
MDA, ng/mg of proteins	3.04 (0.12–21.63)	3.05 (0.22–8.43)	0.935
8-OHdG, ng/mg of proteins	0.11 (0.04–7.92)	0.12 (0.05–0.71)	0.504

SOD = superoxide dismutase; GPx = glutathione peroxidase; GST = glutathione-S transferase, MDA = malonaldehyde; 8OHdG = 8-hydroxy-2′-deoxyguanosine.

## Data Availability

The data supporting the reported results can be provided upon request in the form of datasets available at the Gynecology and Obstetrics Clinic “Narodni Front” and the Institute of Medical and Clinical Biochemistry, Faculty of Medicine, University of Belgrade.

## Supplementary Materials

The supporting information can be downloaded at: https://www.mdpi.com/article/10.3390/ijms26010388/s1.
