# Correction: Jeremic et al. Oxidative Homeostasis in Follicular Fluid and Embryo Quality—A Pilot Study. *Int. J. Mol. Sci.* 2025, *26*, 388

**DOI:** 10.3390/ijms26083560

**Published:** 2025-04-10

**Authors:** Ana Jeremic, Mladenko Vasiljevic, Zeljko Mikovic, Zoran Bukumiric, Petar Simic, Tamara Stanisavljevic, Tatjana Simic, Tatjana Djukic

**Affiliations:** 1 University Clinic for Gynecology and Obstetrics “Narodni Front”, 11000 Belgrade, Serbia; ana.ivf.ana@gmail.com (A.J.); simicp93@gmail.com (P.S.); 2 Faculty of Medicine, University of Belgrade, 11000 Belgrade, Serbia; zoran.bukumiric@med.bg.ac.rs (Z.B.); tamara.stanisavljevic@med.bg.ac.rs (T.S.); 3 Institute of Medical Statistics and Informatics, 11000 Belgrade, Serbia; 4 Institute of Medical and Clinical Biochemistry, 11000 Belgrade, Serbia; 5 Center of Excellence for Redox Medicine, 11000 Belgrade, Serbia; 6 Serbian Academy of Sciences and Arts, 11000 Belgrade, Serbia

There was an error in the original publication [1].

A correction has been made to the Conflicts of Interest statement to reflect the accurate disclosure of potential conflicts. The corrected Conflicts of Interest statement is below.

Conflicts of Interest: The authors would like to disclose a personal relationship: Petar Simic is Tatjana Simic’s son, but this does not entail or encompass any potential conflicts of interest.

The authors state that the scientific conclusions are unaffected. This correction was approved by the Academic Editor. The original publication has also been updated.
