# Supplementary material for: Oxidative Homeostasis in Follicular Fluid and Embryo Quality—A Pilot Study
Source: Int J Mol Sci. 2025 Jan 4;26(1):388. doi: 10.3390/ijms26010388 (PMC11721896; doi:10.3390/ijms26010388)
Supplement: Supplementary file 1 [file ijms-26-00388-s001.zip › ijms-3368232-supplementary.pdf]

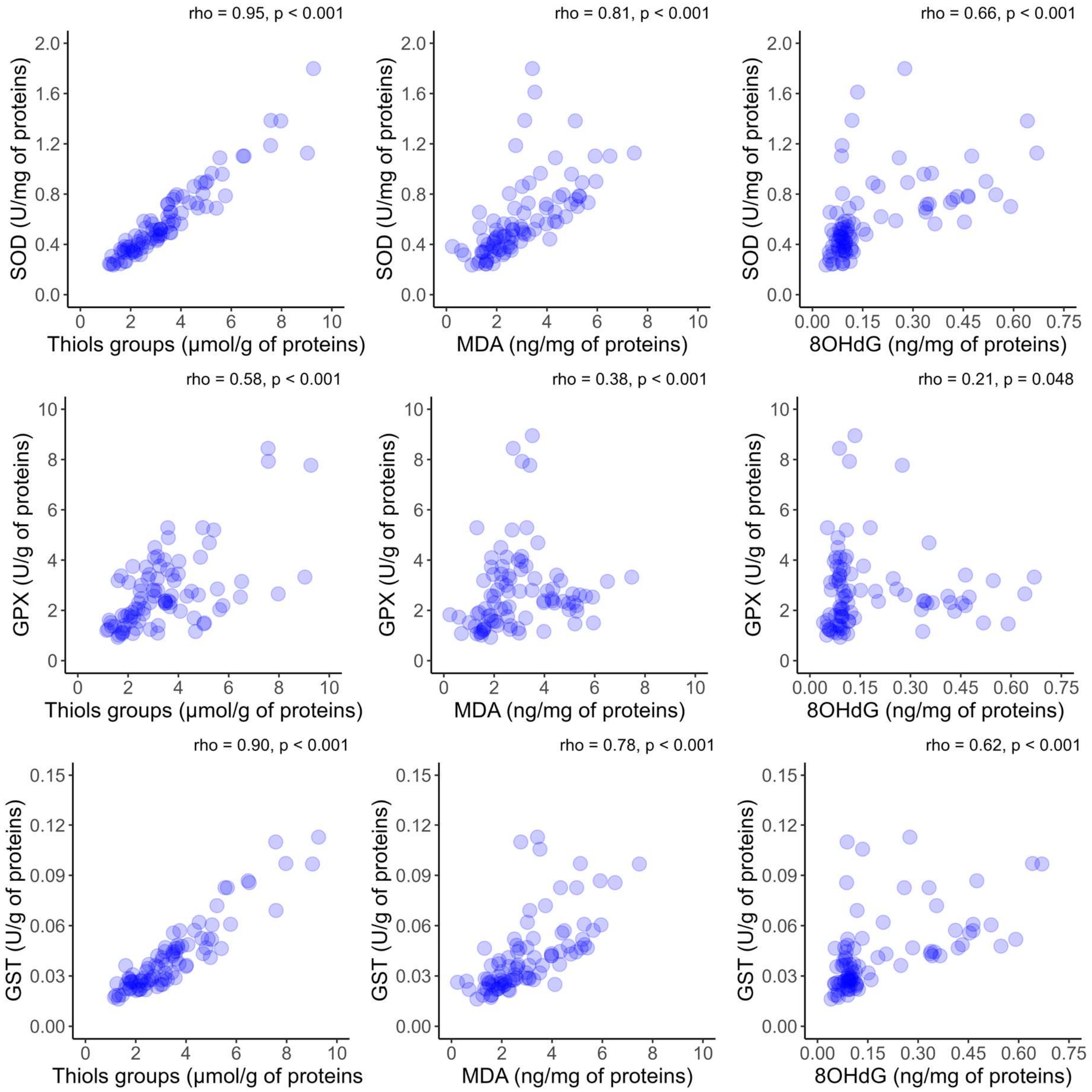

Figure S1: Scatter plot analysis of the correlation between oxidative stress markers and antioxidant enzyme activities in follicular fluid in the group consisting of Grade I embryos. Outliers are not shown in the graph for a better visualization of the correlation. SOD = superoxide dismutase; GPx = glutathione peroxidase; GST = glutathione-S transferase; MDA = malonaldehyde; 8OHdG = 8-hydroxy-2′ -deoxyguanosine.

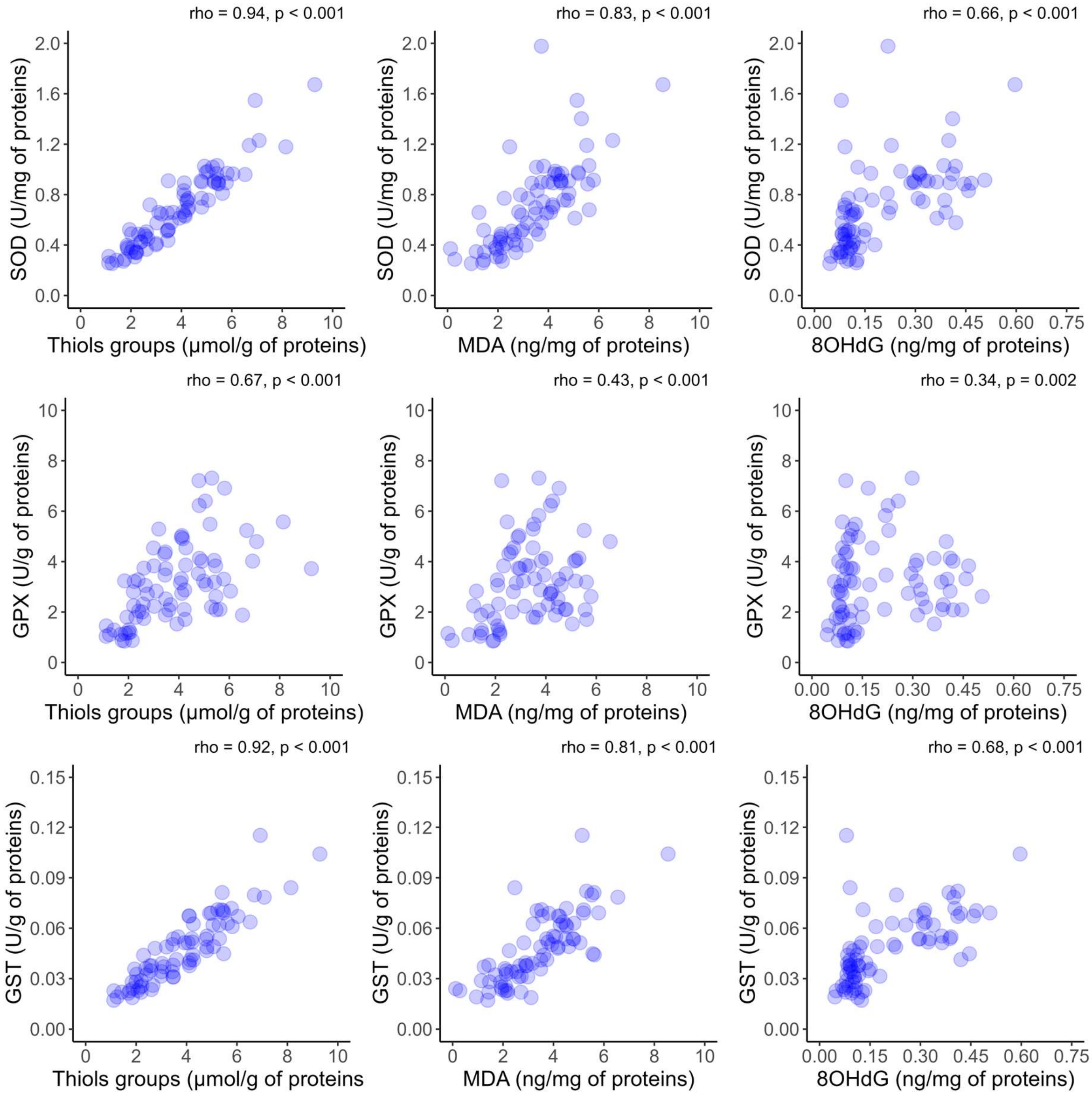

Figure S2: Scatter plot analysis of the correlation between oxidative stress markers and antioxidant enzyme activities in follicular fluid in the group consisting of Grade II-IV embryos. Outliers are not shown in the graph for a better visualization of the correlation. SOD = superoxide dismutase; GPx = glutathione peroxidase; GST = glutathione-S transferase; MDA = malonaldehyde; 8OHdG = 8-hydroxy-2′-deoxyguanosine.
